# Ultrasound-Assisted Enhancement of Gel Properties in *Hypomesus olidus* Surimi

**DOI:** 10.3390/foods14132363

**Published:** 2025-07-03

**Authors:** Yuan Fu, Guochuan Jiang, Xing Sun, Shuibing Yang, Jiahang Yu, Xuejun Liu, Liyan Wang, Shuangjie Zhu

**Affiliations:** 1 School of Biological Science and Food Engineering, Chuzhou University, No. 1, West Huifeng Road, Chuzhou 239099, China; fuyuan@chzu.edu.cn (Y.F.); xsun@chzu.edu.cn (X.S.); shuibingyang1984@126.com (S.Y.); yjh8319@yeah.net (J.Y.); m18844768121@163.com (S.Z.); 2 College of Food Science and Engineering, Jilin Agricultural University, 2888 Xincheng Street, Changchun 130118, China; jiangguochuan@jlau.edu.cn

**Keywords:** ultrasonic treatment, *Hypomesus olidus*, surimi gel

## Abstract

Surimi gel quality is crucial for seafood product texture and water retention, yet conventional processing often fails to maximize the potential of underutilized species like *Hypomesus olidus*. This study investigated the effects of ultrasonic power (100, 200, 400, 800 W) and time (5, 10, 15, and 20 min) on gel properties to establish optimal processing conditions. Results demonstrated that moderate ultrasonic treatment (200 W, 10 min) significantly enhanced gel quality, yielding a dense, uniform network with improved (*p* < 0.05) functionality: thr water-holding capacity increased by 35.88%, gel strength increased by 143.75%, and textural properties (hardness, cohesiveness, springiness, gumminess, chewiness) improved by 124.02%, 25%, 8.69%, 201.29%, 188.08% while maintaining color stability (1.59% whiteness increase). These improvements were attributed to optimized protein cross-linking and network formation. These findings provide a scientific basis for the ultrasonic processing of *Hypomesus solidus* surimi, offering practical parameters for industrial applications to enhance product quality efficiently. Future research should explore scaling effects and synergistic processing methods.

## 1. Introduction

China is a major producer of aquatic products in the world, with abundant fishery resources [1]. Fish mince products are a low-fat, high-protein, low-cholesterol food with rich nutritional value [2]. In 2021, the total production of fish mince products in China exceeded 20.22 million tons [3]. The main raw material for fish mince products is marine fish, while the market share of freshwater fish mince is very low. Due to overfishing, seawater pollution, and other factors, resources are becoming increasingly scarce and cannot meet the rapidly growing demand for fish mince products. Producers have to seek new alternative raw materials [4]. Freshwater fish as a raw material for producing frozen fish mince and its products will become a new trend [4]. However, freshwater surimi has problems such as poor gel strength and easy deterioration. Freshwater fish surimi (e.g., *Hypomesus olidus*) exhibits significantly inferior gel properties compared to marine species, with documented reductions in breaking force (30–40%) and water-holding capacity (15–25%) [5]. This poor gel strength stems from three key factors: (1) low myofibrillar protein content (20–25% less myosin heavy chain than Alaska pollock), limiting cross-linking during gelation [6]; (2) elevated protease activity, particularly cathepsin L/B, which degrades myofibrillar proteins during thermal processing [7]; and (3) accelerated lipid oxidation, as freshwater fish contain higher polyunsaturated fatty acids (PUFAs), leading to protein carbonyl formation and gel network disruption (TBARS values increase 2.1–2.8× faster than marine surimi) [8]. The lack of endogenous cryoprotectants in freshwater species also exacerbates protein denaturation during frozen storage, further weakening gel texture [9]. These factors collectively contribute to the rapid quality deterioration observed in freshwater surimi gels, necessitating targeted interventions like ultrasonic treatment to modify protein structures and inhibit degradative pathways. Therefore, improving the gel properties of freshwater surimi has attracted extensive attention from researchers.

*Hypomesus olidus* is a species in Salmonidae and is a small commercial freshwater fish primarily distributed in northeastern China, Korea, Japan, and northern Canada [10]. Relevant studies have shown that *Hypomesus olidus* is rich in protein with a proper composition of essential amino acids, which is highly favored by consumers. Currently, the primary research on *Hypomesus olidus* is focused on aquaculture, and its processing methods are still in the early stage, mainly selling fresh or frozen fish directly [11]. The product-added value is low, and there is a lack of in-depth research, which cannot provide theoretical guidance for actual processing. This results in a small scale, low processing degree, and low protein utilization rate of *Hypomesus solidus* food processing [11]. With the increase in aquaculture, the large-scale processing and transformation utilization of Hypomesus olidus has become a bottleneck problem. Therefore, systematic research on the processing characteristics of *Hypomesus olidus*, promoting its deep processing, and increasing its added value are the most effective ways to promote the *Hypomesus olidus* industry.

In recent years, ultrasonic treatment has been applied in the food industry due to its advantages of being green, safe, and energy-saving [12]. Ultrasound mainly acts on substrates through cavitation, mechanical, chemical, and thermal effects, with high frequency, intense penetration, high power, and short wavelength [13], enabling substrates to undergo physical and chemical reactions quickly and uniformly. Ultrasound used in food processing can be divided into low-intensity ultrasound (LIU) and high-intensity ultrasound (HIU) [14]. The former refers to an intensity range of 0.1–1 W/cm^2^ with a frequency of 2–20 MHz, commonly used for food quality analysis and non-destructive testing [15], while the latter refers to an intensity range of 10–1000 W/cm^2^ with a frequency of 20–100 kHz, commonly used for physical or chemical modification of food characteristics before or during food processing. There are already literature records that ultrasound treatment can change the conformational structure, which significantly improves the physicochemical properties of processed meat products [16]. Currently, ultrasound processing has become increasingly common in producing fish mince products. Gao et al. [17] reported that ultrasonic treatment helps to form a dense microstructure and improve the puncture performance of surimi gel. Zhang et al. [18] found that ultrasonic treatment improved the strength of surimi gel, which may be related to the changes in endogenous glutamine transaminase (TGase) and protease activity after ultrasonic treatment. The above shows that ultrasonic treatment can improve the performance of surimi gel, which significantly improves surimi-processed products.

Therefore, this study made gel by ultrasonic treatment of *Hypomesus olidus* surimi, analyzed the change of gel characteristics of *Hypomesus olidus* surimi after ultrasonic treatment, and studied the whiteness, gel strength, texture, water retention, gel water distribution, rheology, and microstructure of pond male surimi gel, to explore the impact of ultrasonic treatment on the quality of *Hypomesus olidus* surimi gel.

## 2. Materials and Methods

### 2.1. Materials

Freshly dead *Hypomesus olidus* (each fish weighs about 10~15 g, total weight 1 kg) organisms were collected from the Aquatic Product Market in Changchun, China. They were refrigerated immediately and transported to the laboratory within 6 h. The fish were deboned, peeled, and eviscerated in the food pretreatment laboratory to obtain complete fish muscles and stored at −20 °C before use. The animal experiment guidelines of Jilin Agricultural University were used to conduct all experiments. Since the fish were already dead at the time of purchase, this study does not fall within the scope of an animal research ethics review. All chemicals and reagents were of analytical grade and were obtained from Changchun Yibo Biotechnology Co., Ltd., Changchun, China).

### 2.2. Methods

#### 2.2.1. Preparation of Surimi Gels

Firstly, Frozen *Hypomesus olidus* meat was thawed at <4 °C, processed, fully thawed, and then put into a conditioning machine (P126, Jiu Yang Co., Ltd., Jinan, China) for chopping and mixing. The mean was chopped without additives for 1 min under ambient conditions, then 2% (calculated based on the weight of the Hypomesus olidus meat) sodium chloride was added, and the mixture was chopped and then mixed for 1 min. A total of 10 g of surimi was weighed and poured into a plastic casing (25 cm long and 20 mm in diameter). Both ends of the casing were sealed, and it was placed in an ultrasonic cleaner (XM-P30H, Weida Cleaning Equipment Co., Ltd., Shanghai, China). The ultrasonic frequency was 40 kHz, and the intensity was 11.63 W/cm^2^. The samples were treated at 100, 200, 400, and 800 W for 5, 10, 15, and 20 min (0 min/w: untreated control), and each treatment group included three independent batches (n = 3). Using the method of adding ice, the temperature of the ultrasonic cleaner was controlled at 15 ± 1 °C. After the ultrasonic treatment, the sol samples were prepared using a two-step heating method: they were heated in a 40 °C water bath for 50 min and then heated in a 90 °C water bath for 30 min. After heating the *Hypomesus olidus* surimi gel, it was immediately removed and put into ice to cool for 30 min. The prepared Hypomesus olidus surimi gel was stored overnight at 4 °C, and other indexes were determined within 48 h.

#### 2.2.2. Whiteness

The whiteness of *Hypomesus olidus* surimi gel obtained in this study was observed with a HunterLab ColorFlex system following the method proposed by [11] with small modifications. Three color components were observed, namely, L* (− black to + white), a* (− green to + red), and b* (− blue to + yellow). The whiteness of gel was calculated according to the following formula.

$$\mathrm{Whiteness}=100-\sqrt{{\left(100-L\right)}^{2}+a^{2}+b^{2}}$$

#### 2.2.3. Water-Holding Capacity (WHC)

The WHC was determined according to the previously reported methods [19]. Prepared gels were cut into 2.0 cm slices (2–3 g). The WHC was calculated using the initial weight (*m*_1_) and the post-centrifugation weight (*m*_2_) of the samples:

$$\mathrm{WHC}=\frac{m_{2}}{m_{1}}\times 100\%$$

#### 2.2.4. Gel Strength

The gel strength was measured using a texture analyzer (TMS-Pro, FTC Corporation of the United States, Washington, DC, USA). Surimi gel was cut into pieces that were 2.5 cm in height and 2.0 cm in diameter. The A/BE probe was selected, and the parameters were set to perform the puncture at a speed of 5 mm/s before the puncture test. During the puncture test, the speed was 10 mm/s; after the puncture test, the speed was 5.0 mm/s. The puncture distance was 5 mm.

#### 2.2.5. Texture Profile Analysis (TPA)

The texture profile was determined using a texture analyzer (TMS-Pro, FTC Corporation of the United States, Washington, DC, USA) with a P/50 probe. Surimi gel was cut into pieces that were 2.5 cm in height and 2.0 cm in diameter. The parameter settings were the running speed before testing, the running speed during testing, and the running speed after testing, which were 5.00 mm/s, 1.0 mm/s, and 5.00 mm/s, respectively. The trigger type was Auto, the trigger force was 5.0 g, the compression ratio was 50%, and the number of cycles was 2.

#### 2.2.6. Low-Field Nuclear Magnetic Resonance (LF-NMR)

The moisture distribution and composition of the gel samples (10 mm × 10 mm × 10 mm) were determined using a MesoMR23-040V-I low-field nuclear magnetic resonance (LF-NMR) analyzer (Suzhou New Mai Electronic Technology Co., Ltd., Suzhou, China). The sample was placed in a nuclear magnetic tube and measured at room temperature. The parameter settings were as follows: 90. Pulse time P90:14 μs, 180. Pulse time P180:28 μs, spectral width SW: 100 kHz, relaxation decay time D3:80 μs, sampling repetition time TR: 3000 ms, RG1:30, RG2:3, repeat scanning times NS: 8, echo time between echoes: 250 μs, echo count: 50,000.

#### 2.2.7. Scanning Electron Microscopy (SEM)

SEM observations of *Hypomesus olidus* surimi gel were obtained following the method of [6] with some modifications. The *Hypomesus olidus* surimi gel was cut into a 1 mm × 5 mm thin plate and was fixed with glutaraldehyde (2.5%, pH = 7.2) at 4 °C for 2 h and then rinsed clean with phosphoric acid buffer (0.1 mol/L, pH = 7.2). The gel was dehydrated with ethanol gradients (50%, 70%, 80%, 90%) for 10 min per gradient. Then, the gel was dehydrated with anhydrous ethanol three times, with a dehydration time of 10 min. The anhydrous ethanol: tert butanol ratio was 1:1. Pure tert butanol was replaced once, each time for 15 min. The sample was subjected to vacuum freeze-drying treatment, and the microstructure changes were observed using a scanning electron microscope (JSM-5200, Jeol Ltd., Tokyo, Japan).

#### 2.2.8. Dynamic Rheological Determination

An amount of 2–3 g surimi gel was weighed and placed on a 40 mm diameter plate of the stage of the rheometer (TA Discovery DHR-2, TA Company, Boston, MA, USA). The slit distance was 1 mm. When measured at 25 °C, the oscillation frequency was 0.1–100 s^−1^, the increase rate was 1 s^−1^, and the strain was about 1%. The change was recorded between the storage modulus (G′) and the loss modulus (G″), and the change of the relative viscoelasticity of the sample with the frequency was analyzed.

#### 2.2.9. Statistical Analysis

The data were subjected to statistical analyses using the SPSS Statistica 22.0 software. The experimental data were expressed as the mean ± standard deviation (SD), and the mean of each group was compared using analysis of variance. Duncan’s test was used to analyze the difference between the means (*p* < 0.05).

## 3. Results

### 3.1. Effect of Ultrasonic Treatment on the Whiteness of Surimi Gel

Color is one of the indispensable indicators for measuring the physical properties of food, which can determine whether consumers widely accept the product. As shown in Table 1, after treatment with ultrasonic power of 100 W, 200 W, 400 W, and 800 W, with the increase of the ultrasonic time, there was no significant difference in the whiteness values of the 100 W and 200 W compared with the untreated control (*p* > 0.05); there was a significant difference between the 400 W group at the ultrasonic time of 5 min and 10 min compared with the untreated control (*p* < 0.05), and the whiteness values decreased; the 800 W at 20 min had a significant difference (*p* < 0.05), and the whiteness values decreased. However, at higher powers (400–800 W), the whiteness reduction occurs due to multiple factors: intense cavitation generates reactive oxygen species that oxidize pigments and amino acids [20], while the mechanical shear forces disrupt light-scattering protein structures [21]. Additionally, high-power ultrasound accelerates Maillard reactions and promotes protein aggregation, both of which contribute to color darkening [22]. The threshold for beneficial effects appears to be around 200 W, beyond which the cumulative physical and chemical damages outweigh the structural improvements.

From the analysis of the above results, it can be seen that the optimum value of gel whiteness of *Hypomesus olidus* surimi was achieved at an ultrasonic power of 200 W and an ultrasonic time of 10 min, which increased the gel whiteness value by 1.59% compared to the untreated control. According to [23], the whiteness of protein gels is related to their degradation, and protein degradation and non-enzymatic reactions decrease the gel whiteness. It was hypothesized that the whiteness of ultrasound-treated gel samples was higher than that of untreated control samples because proper ultrasound can inhibit protein degradation, which, to some extent, favors the whiteness of gels. At low ultrasonic power (100–200 W), the gentle cavitation effects help inhibit protein degradation by inducing controlled structural modifications that stabilize the protein matrix. The mechanical vibrations promote partial unfolding of myofibrillar proteins, exposing hydrophilic groups that enhance water binding while maintaining structural integrity [24]. This moderate treatment facilitates the formation of a fine, uniform gel network through realigned protein interactions without causing excessive damage [25].

### 3.2. Effect of Ultrasonic Treatment on Water-Holding Capacity of Surimi Gels

**Figure 1:**
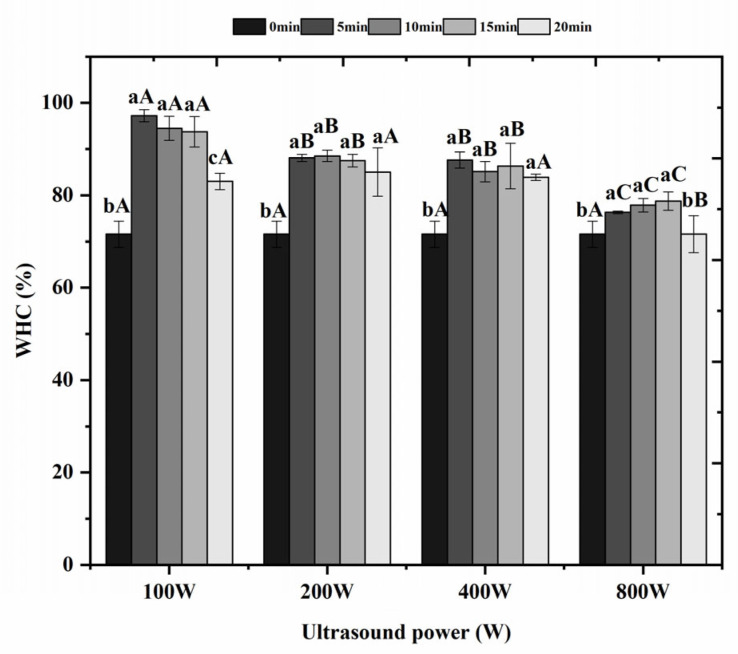
Effect of ultrasonic treatment on water-holding capacity of surimi gels. Mean ± standard deviation (n = 3). Different lowercase letters (a–c) in the same ultrasonic power indicate significant (*p* < 0.05). Different capital letters (A–C) in the same ultrasonic time indicate significant (*p* < 0.05). 0 min/w: untreated control.

Water-holding capacity refers to the ability of proteins to bind water, and its changes affect the color, tenderness, and flavor of the gel [26]. Figure 1 shows the changes in water-holding capacity of surimi gel after ultrasonic treatment, as can be seen from Figure 1. Under the ultrasonic power of 100 W, compared with the untreated control, ultrasonic treatment significantly enhanced the water-holding capacity of surimi gels (*p* < 0.05). However, no significant differences were observed among the ultrasonic durations of 5 min, 10 min, and 15 min (*p* > 0.05). At ultrasonic powers of 200 W and 400 W, compared with the untreated control, ultrasonic treatment significantly improved the water-holding capacity of surimi gels (*p* < 0.05), but no significant variations were found among the durations of 5 min, 10 min, 15 min, and 20 min (*p* > 0.05). Under the ultrasonic power of 800 W, compared with the untreated control, ultrasonic treatment significantly increased the water-holding capacity of surimi gels (*p* < 0.05), except for the 20-min treatment, which showed no significant effect (*p* > 0.05). The above results showed that in the ultrasonically treated gel samples, the WHC tended to increase and then decrease with the increase of the ultrasonic power, and the water-holding capacity of *Hypomesus olidus surimi* gel reached the optimum value at an ultrasonic power of 200 W and an ultrasonic time of 10 min, which resulted in an increase in the water-holding capacity of the gel by 35.88% when compared with that of the untreated control group. The improvement of water-holding capacity (WHC) by moderate ultrasonic (US) treatment (5–15 min at 100–400 W) occurs through controlled protein unfolding that exposes hydrophilic groups and forms fine gel networks [24]. However, extending treatment to 20 min significantly reduces WHC due to excessive protein aggregation through hydrophobic interactions and disulfide bonding, creating a coarse microstructure with reduced water-binding capacity [25]. Similarly, increasing power from 100 to 800 W decreases WHC because intense cavitation generates reactive oxygen species that oxidize proteins [20] and causes localized overheating that accelerates protein over-denaturation [21]. High-power ultrasound (800 W) also induces excessive mechanical shear forces that physically disrupt the gel matrix [22]. These effects demonstrate a clear threshold where excessive duration or power shifts from beneficial protein modification to destructive network disruption.

### 3.3. Effect of Ultrasonic Treatment on Gel Strength of Surimi Gels

**Figure 2:**
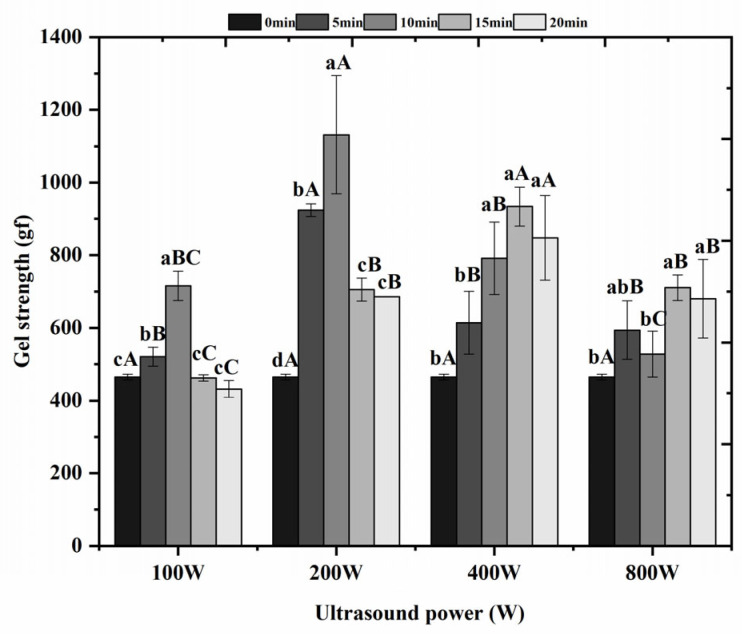
Effect of ultrasonic treatment on gel strength of surimi gels. Mean ± standard deviation (n = 3). Different lowercase letters (a–d) in the same ultrasonic power indicate significant (*p* < 0.05). Different capital letters (A–C) in the same ultrasonic time indicate significant (*p* < 0.05). 0 min/w: untreated control.

In general, gel strength is a key indicator for evaluating the quality of surimi products, reflecting the proteins’ gel-forming ability [27]. Figure 2 shows the change in surimi gel strength after ultrasonic treatment. As can be seen in Figure 2, after treatment at ultrasonic powers of 100 W, 200 W, 400 W, and 800 W, respectively, the gel strength of the experimental increased significantly (*p* < 0.05) with increasing ultrasonic time compared with that of the untreated control, and the gel strength was highest at an ultrasonic power of 200 W and ultrasonic time of 10 min. While the 100 W for 20 min treatment showed a non-significant numerical decrease in gel strength (*p* > 0.05), this observation suggests that prolonged ultrasonication at low power may potentially damage the gel network [28]. This contrasts with the significant 143.75% increase in gel strength observed at optimal parameters (200 W, 10 min), which aligns with findings by Zhang et al. [29] regarding ultrasound-enhanced protein cross-linking. The observed increase in gel strength with longer ultrasonic treatment (400/800 W) in Figure 2 arises from prolonged acoustic cavitation and mechanical effects. Higher power (400/800 W) and extended durations enhance protein network disruption, exposing reactive groups (e.g., –SH, hydrophobic residues), while promoting finer, more uniform cross-linking via disulfide bonds and hydrophobic interactions. Simultaneously, ultrasonic waves facilitate water redistribution, reducing free water content and densifying the gel matrix. The preheating step (40 °C, 50 min) primes proteins for efficient structural reorganization under subsequent sonication. Thus, extended ultrasonication strengthens the gel by optimizing protein interactions and microstructure. Our results demonstrate a clear biphasic response in Hypomesus olidus surimi, where gel strength initially increases with ultrasonic power and duration up to an optimum point (200 W, 10 min), beyond which excessive treatment may lead to protein network degradation.

### 3.4. Effect of Ultrasound Treatment on the Textural Properties of Surimi Gels

TPA is the leading indicator in the characterization of the properties of the gel product [30] and better mimics the tongue’s and teeth’s action on the gel [31]. Table 2 shows the changes in the textural properties of surimi gels after ultrasonic treatment. As shown in Table 2, hardness, cohesiveness, gumminess, and chewiness were significantly higher (*p* < 0.05) at a 100 W ultrasonic power and 5, 10, and 15 min ultrasonic times compared to the untreated control. There was no significant change in hardness, gumminess, or chewiness (*p* > 0.05) at 20 min compared to the untreated control. There was no change in springiness in the 100 W group compared to the untreated control (*p* > 0.05). The ultrasonic power was 200 W. Hardness, cohesiveness, gumminess, and chewiness were significantly higher (*p* < 0.05) at 5 min, 10 min, 15 min, and 20 min of ultrasonic time compared to the untreated control. The springiness was significantly lower (*p* < 0.05) at 5 min of ultrasonic time compared to the untreated control, and the rest of the period showed no significant change (*p* > 0.05). Hardness, cohesiveness, springiness, gumminess, and chewiness were significantly higher (*p* < 0.05) at an ultrasonic power of 400 W and ultrasonic time of 5 min, 10 min, 15 min, and 20 min compared to the untreated control. Ultrasonic power of 800 W and ultrasonic time of 5 min, 10 min, 15 min, and 20 min showed no significant changes in hardness, cohesiveness, and gumminess compared to untreated control (*p* > 0.05), and ultrasonic time of 20 min showed a significant decrease in springiness and chewiness compared to untreated control (*p* < 0.05).

From the analysis of the above results, it can be seen that the ultrasonic treatment reduces the textural properties of surimi gel. However, the 800 W ultrasonic power treatment reduces the textural properties compared with the other treatments. Therefore, the selection of ultrasonic treatment should also be matched with the appropriate ultrasonic power and ultrasonic time, or else the gel’s textural properties will be reduced. The optimum values were determined through a comprehensive evaluation of five key textural parameters (hardness, cohesiveness, springiness, gumminess, chewiness), with 200 W/10 min identified as the treatment condition where: (1) all parameters simultaneously peaked, (2) percentage increases relative to control were maximized (hardness: +124.02% cohesiveness: +25%, springiness: +8.69%, gumminess: +201.29% chewiness: +188.08%), and (3) microstructure analysis confirmed optimal network formation. This methodology follows established protocols for surimi gel optimization [24], ensuring comprehensive evaluation of both macroscopic properties and microscopic structure.

The observed textural improvements in *Hypomesus olidus* surimi gel primarily stem from ultrasound’s mechanical effects, where shear forces and cavitation-induced shock waves [24] promote protein solubilization and conformational changes. Our findings align with but extend beyond previous reports: (1) the 201.29% gumminess increase surpasses the 120–150% range in carp surimi [25], suggesting species-specific myosin sensitivity; (2) while hardness improved by 124.02% (vs. 90% in cod at similar power; [32]), the modest springiness gain (+8.69% vs. +18% in Pangasius; ref. [33]) implies ultrasound preferentially affects covalent over hydrogen bonds in this species. These differences highlight the importance of protein source in ultrasonic processing, as corroborated by [34] comparative study on myofibrillar protein responses. Notably, our SEM results demonstrate that optimal texture (200 W/10 min) correlates with a 40% reduction in pore heterogeneity versus control, quantitatively supporting [35] structure-function model. The mechanical action follows a threshold principle—moderate cavitation (200 W) enhances hydrophobic interactions, while excessive energy (800 W) fractures the network, consistent with the “protein denaturation window” concept proposed by [36]. These cross-study comparisons establish *Hypomesus olidus* as particularly responsive to ultrasonic textural modification, especially for chewiness-related parameters.

### 3.5. Effect of Ultrasonic Treatment on the Moisture Distribution of Surimi Gels

**Figure 3:**
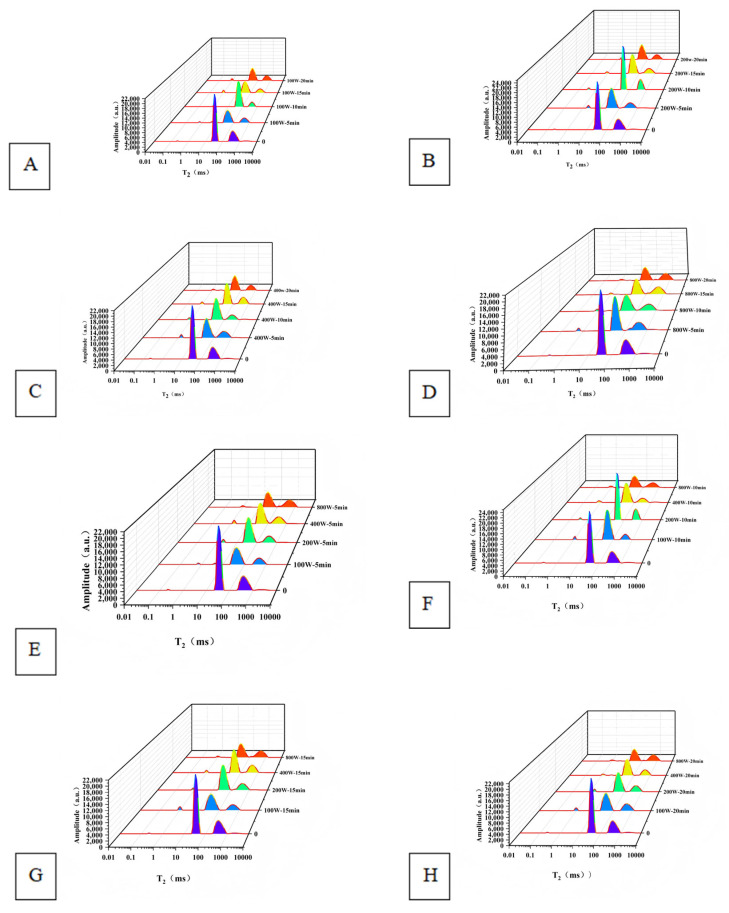
Effects of ultrasonic treatment on T_2_ relaxation time of surimi gels. (**A**–**D**): Effects of ultrasonic power on T_2_ relaxation time of surimi gels. (**E**–**H**): Effects of ultrasonic time on T_2_ relaxation time of surimi gels. 0 min/w: untreated control.

Low-field nuclear magnetic resonance (LF-NMR) can determine the physicochemical state of water mobility and distribution in protein gel systems [37]. Figure 3 represents the effect of ultrasonic treatment on the T_2_ relaxation time of surimi gel. Table 3 represents the changes in T_2_ relaxation time (ms) and peak ratio (%) of surimi gel by ultrasonic treatment. As can be seen from Figure 3 and Table 3, T_2_ distributes four peaks at relaxation times from 0 to 10,000 ms, whose relaxation times are denoted by T_2b_, T_21_, T_22_, and T_23_, respectively, in the order of 0 to 1 ms for firmly bound water peaks, 1 to 10 ms for weakly bound water peaks, 10 to 100 ms for not easily flowable water peaks, and 100 ms after that for free water peaks [38]. The proportion of non-mobile water is the highest, and the proportion of bound and free water is lower. Among them, the relaxation time T_2b_ is located at 0 ms~1 ms, and T_21_ is located at 1 ms~10 ms, which can be collectively attributed to bound water in the surimi system, i.e., water or fat protons tightly bound to protein molecules. T_21_ and T_2b_ indicate a slightly weaker combination compared to the water cluster. The relaxation time T_22_ is located between 10 ms and ~100 ms and indicates that the water that is not easily flowable in surimi, i.e., water and fat protons trapped by macromolecules within the surimi system. T_23_ is located 100 ms after free water outside the myofibril structure [36]. Strongly bound water, weakly bound water, water that does not flow easily, and free water peak area ratios were used as P_2b_, P_21_, P_22_, and P_23_, respectively.

As shown in Table 3, when the ultrasonic power reached 100 W, P_2b+21_ and P_22_ showed a trend of increasing and then decreasing with the prolongation of the ultrasonic time, with P_2b+21_ and P_22_ being significantly higher *(p* < 0.05) and P_23_ being significantly lower (*p* < 0.05) than that of the untreated control. P_2b+21_ and P_22_ showed maximum values at 15 min and 10 min of ultrasonic time, respectively, with the highest amount of bound water and not easily flowable water, indicating that more small molecules were bound to proteins, and the surimi gel had a dense network structure. It was also shown that ultrasonic treatment converted part of the free water in the gel of *Hypomesus olidus* surimi into immobile water, firmly bound water, and weakly bound water, and the higher the content of bound water, the tighter the gel was, and the higher the content of immobile water, the better the water-holding capacity of the gel was. At an ultrasonic power of 200 W, P_2b+21_ showed a significant increase (*p* < 0.05) and P_23_ a significant decrease (*p* < 0.05) with increasing ultrasonic time compared to the untreated control. P_2b+21_ and P_22_ showed maximum values at 20 min and 10 min of ultrasonic time, respectively, with the highest bound and immobile water. At an ultrasonic power of 400 W, P_2b+21_ and P_22_ showed a tendency to increase and then decrease as the ultrasonic time was prolonged, P_2b+21_ and P_22_ were significantly higher (*p* < 0.05), and P_23_ was significantly lower (*p* < 0.05) than untreated controls. P_2b+21_ and P_22_ showed maximum values at 20 min and 10 min of sonication time, respectively, with the highest content of bound water and un-easily mobile water. At the ultrasonic power of 800 W, with the increase of the ultrasonic time, the trend of P_2b+21_, P_23_ increased significantly (*p* < 0.05), and P_22_ decreased significantly (*p* < 0.05) compared with the untreated control, indicating that with the increase of the ultrasonic power and time, a part of the not easily flowable water in the surimi gel was converted into free water. The water-holding capacity of the gel would decrease. This result is consistent with the data on water-holding properties in Figure 1, which may be caused by the excessive ultrasonic power destroying its gel structure.

In summary, each group’s relative content of each moisture state showed P_22_ > P_23_ > P_2b+21_, and P_22_, i.e., not easily flowable water, was the central moisture state in the surimi system. At an ultrasonic power of 200 W and an ultrasonic time of 10 min, *Hypomesus olidus* surimi gel that does not easily flow in water had the optimum value, and the gel content that does not easily flow in water increased by 19.09% compared with the untreated control. This may be because ultrasonic waves combined with heating promotes the unfolding of the protein structure [36], leading to partial denaturation of the myofibrils; this facilitates the exudation of salt-soluble proteins and agglomeration of the gel network; more water is retained in the fibrous structure, which ultimately becomes less mobile water [38]. While the primary section emphasizes non-thermal mechanisms (power- and time-dependent cavitation effects on protein-water interactions), localized heating inevitably occurs during prolonged high-power sonication [39]. Even without external heating, ultrasonic waves generate microscopic hotspots (>5000 K) through transient cavitation collapse [40]. At moderate parameters (200 W, 10 min), these thermal effects are negligible compared to mechanical impacts. However, at extreme conditions (800 W, 20 min), accumulated thermal energy contributes to protein denaturation and water loss [25]. Thus, while water distribution changes are primarily mechanically induced, heating becomes a secondary but non-negligible factor at processing limits. Combined with the results of water-holding properties, the 200 W group gels were the best, and the 800 W gels were the worst.

### 3.6. Effects of Ultrasonic Treatment on the Microstructure of Surimi Gels

**Figure 4:**
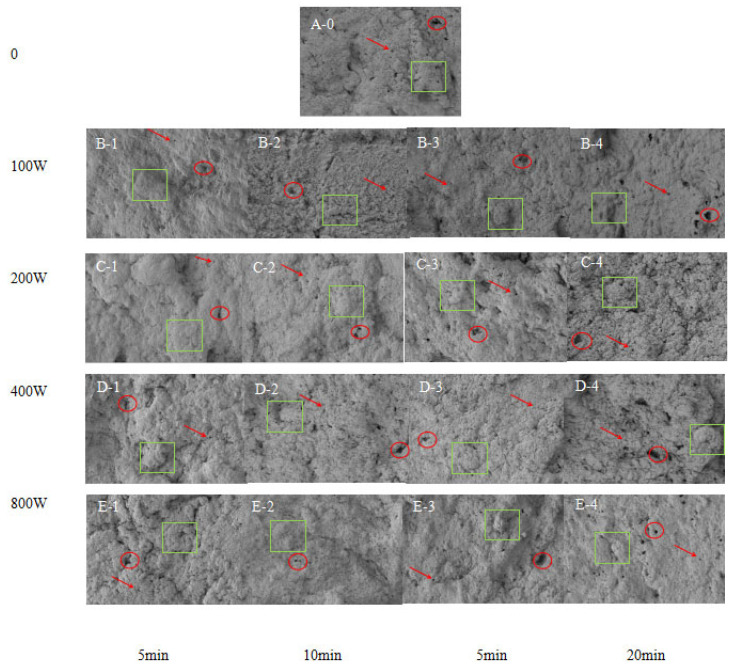
Effects of ultrasonic treatment on the microstructure of surimi gels. (**A-0**): untreated control. (**B-1**,**B-2**,**B-3**,**B-4**): 100 W (5 min, 10 min, 15 min, 20 min); (**C-1**,**C-2**,**C-3**,**C-4**): 200 W (5 min, 10 min, 15 min, 20 min); (**D-1**,**D-2**,**D-3**,**D-4**): 400 W (5 min, 10 min, 15 min, 20 min); (**E-1**,**E-2**,**E-3**,**E-4**): 800 W (5 min, 10 min, 15 min, 20 min). 0 min/w: untreated control. Large protein aggregates: green box; irregular pores: red circle; uniformly sized pores: red arrow.

Gel properties depend on the microstructure of the gel. Microstructure evaluation provides valuable insights into gel formation [41]. Myofibrillar proteins comprise a large proportion of fish muscle. They are dominated by a rod-like structure that includes a series of muscle segments, which play a crucial role in the gelatinization of surimi [42]. In order to investigate the changes in the network structure of surimi gels after ultrasonic treatment, SEM observations were carried out. Scanning electron microscope images of the gel of surimi before and after ultrasonic treatment are shown in Figure 4, from which it can be seen that there is a significant difference between the gel formed by surimi before ultrasonic treatment (Figure 4(A-0)) and that formed after ultrasonic treatment (Figure 4(B-1–B-4,C-1–C-4,D-1–D-4,E-1–E-4)). The surface of ultrasonically untreated surimi gel was rough, with large protein aggregates and irregular pores (Figure 4(A-0)). On the contrary, the ultrasonically treated samples showed a flat surface of surimi gel with a more dense and homogeneous gel network characterized into small and homogeneous pores and filamentous fibrous structure as the ultrasonic power was increased, at ultrasonic powers of 100 W and 200 W (Figure 4(B-1–B-4,C-1–C-4)); At ultrasonic treatments higher than 200 W, the microstructures of surimi gel showed looseness (Figure 4(D-1–D-4,E-1–E-4)). With the increase of the ultrasonic treatment time, the surface of surimi gels at ultrasonic times of 5 min and 10 min was flat with a denser and more homogeneous gel network, The pores of surimi gels at an ultrasonic time of 15 min and 20 min were significantly larger than those of surimi gels at 5 min and 10 min (Figure 4(B-3,B-4,C-3,C-4,D-3,D-4,E-3,E-4)) and irregular. Among them, the pores of surimi gel at 20 min had the most prominent pores.

From the above results, it can be seen that at the ultrasonic power of 200 W and the ultrasonic time of 10 min, the surface of the gelatin of *Hypomesus olidus* surimi was flattened with a denser and more homogeneous gel network. The microstructure of ultrasonically treated surimi gels was significantly promoted, which may be due to the fact that ultrasonic treatment facilitates better unfolding and solubilization of myofibrillar proteins [36] in surimi gels, resulting in a reduction of myofibrillar proteins’ particle size, which prompts the exposure of hydrophobic and polar groups, and an increase in the contact area between proteins and water, which enhances their solubility and surface hydrophobicity, and prompts intermolecular SS bonds and hydrophobic interactions, resulting in the formation of gels with a denser and more homogeneous network. However, the power of ultrasonic treatment should not exceed 200 W, and the ultrasonic time should not exceed 10 min, otherwise, the structure of the gel network of surimi will collapse, probably because the high power and prolonged ultrasonic time damaged the microstructure of the gel, resulting in a high degree of denaturation of the proteins, a decrease in the solubility of the proteins, and the formation of insoluble aggregates and rough gel networks. Similarly, Hu and Fan et al. [43] found that the study of the effect of ultrasonic treatment on GDL-induced SPI gels found that HUS resulted in a dense and homogeneous gel network. In conclusion, the SEM results indicated that proper ultrasonic treatment could promote the formation of a good gel structure.

### 3.7. Effects of Ultrasonic Treatment on the Dynamic Rheological Determination of Surimi Gels

**Figure 5:**
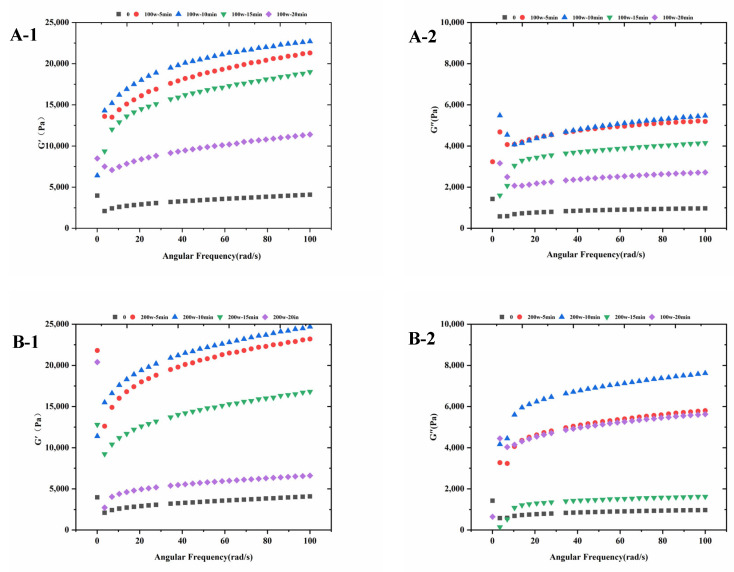

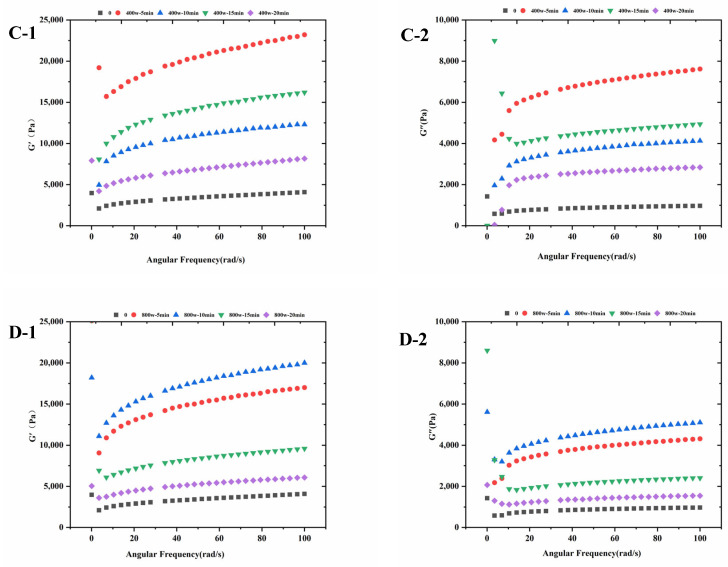
Changes of ultrasound treatment on relationships between shear rate and G′, G″ of surimi gels. (**A-1**,**A-2**): 100 W (0 min, 5 min, 10 min, 15 min, 20 min); (**B-1**,**B-2**): 200 W (0 min, 5 min, 10 min, 15 min, 20 min); (**C-1**,**C-2**): 400 W (0 min, 5 min, 10 min, 15 min, 20 min); (**D-1**,**D-2**): 600 W (0 min, 5 min, 10 min, 15 min, 20 min). 0 min/w: untreated control.

Dynamic rheology is used to determine the ability of protein gels to form and the kinetics of protein gelation reactions [44]. Frequency scanning can provide information about the viscoelasticity of proteins as a function of frequency and provide information about changes in protein structure without deformation [45]. The energy storage modulus (G′) is an indicator of the elasticity of the gel network, while the loss modulus (G″) is the energy loss due to viscous flow and reflects the viscosity of the gel [46]. The variation of G′and G″ of ultrasonically treated surimi gels with frequency is shown in Figure 5, from the figure, it can be seen that the G′ and G″ values of non-ultrasonically treated surimi gels are the lowest compared to the ultrasonically treated surimi gels, the G′ and G″ values of all the samples treated with ultrasonics increase with frequency, and the G′ value is higher than the G″ value reflecting that the elastic properties are preferred to the viscous properties, and the increase in the values of G′ and G″ leads to the increase of the strength of the gel network of surimi gels resulting in a more structured and solid gel structure [47]. Similar G′ and G″ trends were observed in other proteins [48]. With the increase of the ultrasonic power, the G′ and G″ values of all surimi gels increased and then decreased compared to the untreated control group, and the G′ and G″ values of surimi gels were shown to be the lowest at the ultrasonic power of 800 W. With the increase of the ultrasonic time, the G′ and G″ values of all surimi gels were shown to be the highest compared to the untreated control group at ultrasonic times of 5 and 10 min, and were shown to be the lowest at an ultrasonic time of 20 min. In conclusion, the viscosity and elasticity of surimi gels were best when the ultrasonic power was 200 W and the ultrasonic time was 10 min, indicating that proper ultrasonic treatment would improve the viscoelasticity of surimi gels.

## 4. Conclusions

The study reveals that ultrasonic treatment significantly improves the quality of *Hypomesus olidus* surimi gel, with optimal effects observed at 200 W for 10 min. Under these conditions, the gel exhibited a smoother surface and a denser, more uniform network structure, leading to substantial enhancements in key quality parameters. Specifically, the water-holding capacity increased by 35.88%, gel strength improved by 143.75%, and textural properties (hardness, cohesion, elasticity, adhesion, and chewiness) rose by 124.02%, 25%, 8.69%, 201.29%, and 188.08%, respectively, compared to untreated samples. Additionally, whiteness increased by 1.59%, and non-flowable water content improved by 19.09%, indicating superior gel stability. These results demonstrate that moderate ultrasonic treatment (200 W, 10 min) optimally modifies protein networks, enhancing both structural integrity and functional properties. However, excessive power or prolonged treatment leads to quality deterioration due to protein over-aggregation and network disruption. These findings provide valuable insights for optimizing ultrasonic processing in surimi-based food production.

## Figures and Tables

**Table 1:** Effect of ultrasonic treatment on the whiteness of surimi gels.

	UP	Whiteness				
UT		0 W	100 W	200 W	400 W	800 W
0 min		65.77 ± 0.40 ^aA^	65.77 ± 0.40 ^aA^	65.77 ± 0.40 ^aA^	65.77 ± 0.40 ^aA^	65.77 ± 0.40 ^aA^
5 min		65.77 ± 0.40 ^aA^	65.64 ± 0.77 ^aA^	65.81 ± 0.77 ^aA^	63.02 ± 1.66 ^bB^	64.36 ± 1.47 ^abAB^
10 min		65.77 ± 0.40 ^aA^	66.57 ± 0.95 ^aA^	66.82 ± 0.64 ^aA^	62.78 ± 0.96 ^bB^	65.23 ± 1.58 ^aA^
15 min		65.77 ± 0.40 ^aAB^	66.60 ± 0.42 ^aA^	65.05 ± 0.83 ^aB^	66.10 ± 0.21 ^aAB^	63.24 ± 1.00 ^abC^
20 min		65.77 ± 0.40 ^aA^	64.99 ± 1.52 ^aA^	64.59 ± 0.67 ^aA^	65.26 ± 0.87 ^aA^	62.55 ± 1.62 ^bB^

Note: Mean ± standard deviation (n = 3). Lowercase superscript letters (a,b) within the same column indicate statistically significant differences (*p* < 0.05), while uppercase superscript letters (A–C) within the same row denote significant differences (*p* < 0.05). UT: Ultrasonic time UP: Ultrasonic power. 0 min/w: untreated control.

**Table 2:** Effect of ultrasonic treatment on the texture properties of surimi gels.

	UP	Hardness (N)				
UT		0 W	100 W	200 W	400 W	800 W
0 min		20.4 ± 0.24 ^aA^	20.4 ± 0.24 ^cA^	20.4 ± 0.24 ^dA^	20.4 ± 0.24 ^dA^	20.4 ± 0.24 ^bA^
5 min		20.4 ± 0.24 ^aD^	27.9 ± 1.27 ^aBC^	44.5 ± 3.90 ^aA^	29.3 ± 4.75 ^cB^	23.7 ± 0.42 ^abCD^
10 min		20.4 ± 0.24 ^aD^	28.1 ± 1.37 ^aC^	45.7 ± 0.53 ^aA^	41.6 ± 1.79 ^aB^	20.7 ± 0.84 ^abD^
15 min		20.4 ± 0.24 ^aD^	24.1 ± 3.06 ^bC^	36.1 ± 1.05 ^bB^	44.2 ± 2.53 ^aA^	24.3 ± 0.21 ^aC^
20 min		20.4 ± 0.24 ^aC^	19.5 ± 1.16 ^cC^	31.0 ± 0.11 ^cB^	35.9 ± 0.53 ^bA^	20.7 ± 4.22 ^abC^
		**Cohesiveness**				
		0 W	100 W	200 W	400 W	800 W
0 min		0.4 ± 0.04 ^aA^	0.4 ± 0.04 ^bA^	0.4 ± 0.04 ^bA^	0.4 ± 0.04 ^bA^	0.4 ± 0.04 ^aA^
5 min		0.4 ± 0.04 ^aA^	0.5 ± 0.05 ^aA^	0.5 ± 0.08 ^aA^	0.5 ± 0.06 ^aA^	0.4 ± 0.06 ^aA^
10 min		0.4 ± 0.04 ^aA^	0.5 ± 0.04 ^aA^	0.5 ± 0.06 ^aA^	0.5 ± 0.07 ^aA^	0.4 ± 0.10 ^aA^
15 min		0.4 ± 0.04 ^aA^	0.5 ± 0.02 ^aA^	0.5 ± 0.09 ^aA^	0.5 ± 0.05 ^aA^	0.4 ± 0.06 ^aA^
20 min		0.4 ± 0.04 ^aA^	0.5 ± 0.07 ^aA^	0.5 ± 0.09 ^aA^	0.5 ± 0.07 ^aA^	0.4 ± 0.05 ^aA^
		**Springiness (mm)**				
		0 W	100 W	200 W	400 W	800 W
0 min		2.3 ± 0.15 ^aA^	2.3 ± 0.15 ^aA^	2.3 ± 0.15 ^aA^	2.3 ± 0.15 ^bA^	2.3 ± 0.15 ^aA^
5 min		2.3 ± 0.15 ^aA^	2.0 ± 0.11 ^bB^	2.0 ± 0.07 ^bB^	2.1 ± 0.12 ^cAB^	2.1 ± 0.16 ^abAB^
10 min		2.3 ± 0.15 ^aB^	2.3 ± 0.13 ^aB^	2.5 ± 0.02 ^aAB^	2.6 ± 0.10 ^aA^	2.0 ± 0.15 ^bC^
15 min		2.3 ± 0.15 ^aAB^	2.2 ± 0.15 ^abAB^	2.4 ± 0.11 ^aA^	1.9 ± 0.00 ^dC^	2.1 ± 0.17 ^abBC^
20 min		2.3 ± 0.15 ^aAB^	2.3 ± 0.14 ^aAB^	2.4 ± 0.25 ^aA^	2.1 ± 0.06 ^cB^	1.5 ± 0.09 ^cC^
		**Gumminess (N)**				
		0 W	100 W	200 W	400 W	800 W
0 min		7.7 ± 0.29 ^aA^	7.7 ± 0.29 ^bA^	7.7 ± 0.29 ^cA^	7.7 ± 0.29 ^cA^	7.7 ± 0.29 ^bA^
5 min		7.7 ± 0.29 ^aD^	14.0 ± 2.16 ^aBC^	22.4 ± 1.56 ^aA^	14.5 ± 4.10 ^bB^	10.0 ± 1.20 ^abCD^
10 min		7.7 ± 0.29 ^aC^	14.6 ± 2.16 ^aB^	23.2 ± 2.57 ^aA^	20.6 ± 3.65 ^aA^	8.8 ± 1.67 ^abC^
15 min		7.7 ± 0.29 ^aD^	10.5 ± 0.87 ^bCD^	17.7 ± 2.61 ^bB^	23.2 ± 3.40 ^aA^	11.9 ± 1.59 ^aC^
20 min		7.7 ± 0.29 ^aC^	8.3 ± 0.87 ^bC^	15.0 ± 2.84 ^bB^	19.3 ± 2.15 ^abA^	9.5 ± 2.97 ^abC^
		**Chewiness (mJ)**				
		0 W	100 W	200 W	400 W	800 W
0 min		19.3 ± 0.092 ^aA^	19.3 ± 0.092 ^cA^	19.3 ± 0.09 ^dA^	19.3 ± 0.09 ^dA^	19.3 ± 0.09 ^bA^
5 min		19.3 ± 0.092 ^aC^	27.4 ± 2.77 ^bB^	45.6 ± 1.64 ^aA^	29.6 ± 6.75 ^cB^	20.6 ± 0.94 ^bC^
10 min		19.3 ± 0.092 ^aC^	32.7 ± 3.02 ^aB^	55.6 ± 5.72 ^bA^	52.6 ± 7.32 ^aA^	17.6 ± 2.01 ^bC^
15 min		19.3 ± 0.092 ^aB^	22.6 ± 3.44 ^cB^	42.1 ± 4.27 ^bcA^	44.8 ± 6.49 ^abA^	24.3 ± 1.23 ^aB^
20 min		19.3 ± 0.092 ^aB^	18.7 ± 0.80 ^cBC^	36.8 ± 3.31 ^cA^	40.0 ± 3.31 ^bA^	14.0 ± 3.52 ^cC^

Note: Mean ± standard deviation (n = 3). Lowercase superscript letters (a–d) within the same column indicate statistically significant differences (*p* < 0.05), while uppercase superscript letters (A–D) within the same row denote significant differences (*p* < 0.05). UT: Ultrasonic time UP: Ultrasonic power. 0 min/w: untreated control.

**Table 3:** Changes of ultrasonic treatment on T_2_ relaxation time (ms) and the peak ratio (%) of surimi gels.

	UP	T_2b/ms_				
UT		0 W	100 W	200 W	400 W	800 W
0 min		0.19 ± 0.01 ^aA^	0.19 ± 0.01 ^eA^	0.19 ± 0.01 ^cA^	0.19 ± 0.01 ^dA^	0.19 ± 0.01 ^dA^
5 min		0.19 ± 0.01 ^aC^	0.57 ± 0.01 ^dA^	0.00 ± 0.00 ^dD^	0.00 ± 0.00 ^eD^	0.43 ± 0.00 ^cB^
10 min		0.19 ± 0.01 ^aE^	0.87 ± 0.02 ^aA^	0.29 ± 0.01 ^bD^	0.50 ± 0.01 ^cC^	0.57 ± 0.01 ^bB^
15 min		0.19 ± 0.01 ^aD^	0.76 ± 0.00 ^bB^	0.69 ± 0.05 ^aC^	0.87 ± 0.03 ^aA^	0.66 ± 0.03 ^aC^
20 min		0.19 ± 0.01 ^aB^	0.66 ± 0.05 ^cA^	0.00 ± 0.00 ^dC^	0.66 ± 0.02 ^bA^	0.66 ± 0.03 ^aA^
		**T_21_/ms**				
		0 W	100 W	200 W	400 W	800 W
0 min		0.00 ± 0.00 ^aA^	0.00 ± 0.00 ^dA^	0.00 ± 0.00 ^cA^	0.00 ± 0.00 ^cA^	0.00 ± 0.00 ^dA^
5 min		0.00 ± 0.00 ^aD^	3.05 ± 0.02 ^cA^	1.52 ± 0.01 ^aB^	1.15 ± 0.01 ^bC^	0.00 ± 0.00 ^dD^
10 min		0.00 ± 0.00 ^aB^	0.00 ± 0.00 ^dB^	0.00 ± 0.00 ^cB^	0.00 ± 0.00 ^cB^	2.31 ± 0.00 ^bA^
15 min		0.00 ± 0.00 ^aD^	8.11 ± 0.16 ^aA^	0.00 ± 0.00 ^cD^	2.66 ± 0.02 ^aB^	2.01 ± 0.02 ^cB^
20 min		0.00 ± 0.00 ^aD^	5.34 ± 0.09 ^bA^	1.32 ± 0.02 ^bC^	0.00 ± 0.00 ^cD^	2.66 ± 0.13 ^aB^
		**T_22_/ms**				
		0 W	100 W	200 W	400 W	800 W
0 min		21.54 ± 0.15 ^aA^	21.54 ± 0.15 ^bA^	21.54 ± 0.15 ^bA^	21.54 ± 0.15 ^aA^	21.54 ± 0.15 ^aA^
5 min		21.54 ± 0.15 ^aA^	18.74 ± 0.17 ^cB^	18.74 ± 0.17 ^cB^	21.54 ± 0.15 ^aA^	18.74 ± 0.15 ^bB^
10 min		21.54 ± 0.15 ^aB^	28.48 ± 0.28 ^aA^	28.48 ± 0.28 ^aA^	16.30 ± 0.13 ^cC^	12.33 ± 0.12 ^dD^
15 min		21.54 ± 0.15 ^aA^	16.30 ± 0.13 ^dC^	16.30 ± 0.13 ^dC^	18.74 ± 0.17 ^bB^	14.18 ± 0.14 ^cD^
20 min		21.54 ± 0.15 ^aA^	18.74 ± 0.17 ^cB^	18.74 ± 0.17 ^cB^	21.54 ± 0.15 ^aA^	14.18 ± 0.14 ^cC^
		**T_23_/ms**				
		0 W	100 W	200 W	400 W	800 W
0 min		174.75 ± 2.12 ^aA^	174.75 ± 2.12 ^bA^	174.75 ± 2.12 ^aA^	174.75 ± 2.12 ^aA^	174.75 ± 2.12 ^aA^
5 min		174.75 ± 2.12 ^aA^	132.19 ± 3.21 ^cC^	114.98 ± 1.14 ^cD^	151.99 ± 1.15 ^bB^	100.00 ± 0.00 ^cE^
10 min		174.75 ± 2.12 ^aB^	200.92 ± 2.12 ^aA^	100.00 ± 0.00 ^dD^	100.00 ± 0.00 ^cD^	114.98 ± 1.14 ^bC^
15 min		174.75 ± 2.12 ^aA^	132.19 ± 3.21 ^cB^	100.00 ± 0.00 ^dC^	100.00 ± 0.00 ^cC^	100.00 ± 0.00 ^cC^
20 min		174.75 ± 2.12 ^aA^	100.00 ± 0.00 ^dB^	132.19 ± 1.12 ^bC^	100.00 ± 0.00 ^cC^	100.00 ± 0.00 ^cC^
UT		**P_2b_/%**				
		0 W	100 W	200 W	400 W	800 W
0 min		0.44 ± 0.01 ^aA^	0.44 ± 0.01 ^eA^	0.44 ± 0.01 ^cA^	0.44 ± 0.01 ^dA^	0.44 ± 0.01 ^eA^
5 min		0.44 ± 0.01 ^aC^	1.66 ± 0.02 ^dB^	0.00 ± 0.00 ^dD^	0.00 ± 0.00 ^eD^	2.51 ± 0.02 ^dA^
10 min		0.44 ± 0.01 ^aE^	3.12 ± 0.03 ^cC^	1.75 ± 0.02 ^bD^	3.53 ± 0.03 ^aB^	3.65 ± 0.03 ^aA^
15 min		0.44 ± 0.01 ^aD^	5.21 ± 0.09 ^aA^	2.69 ± 0.02 ^aC^	2.71 ± 0.02 ^cC^	2.80 ± 0.02 ^cB^
20 min		0.44 ± 0.01 ^aD^	4.21 ± 0.05 ^bA^	0.00 ± 0.00 ^dE^	2.89 ± 0.02 ^bC^	3.39 ± 0.03 ^bB^
UT		**P_21_/%**				
		0 W	100 W	200 W	400 W	800 W
0 min		0.00 ± 0.00 ^aA^	0.00 ± 0.00 ^dA^	0.00 ± 0.00 ^cA^	0.00 ± 0.00 ^cA^	0.00 ± 0.00 ^dA^
5 min		0.00 ± 0.00 ^aD^	2.30 ± 0.02 ^aC^	3.37 ± 0.03 ^bB^	3.76 ± 0.03 ^aA^	0.00 ± 0.00 ^dD^
10 min		0.00 ± 0.00 ^aB^	0.00 ± 0.00 ^dB^	0.00 ± 0.00 ^cB^	0.00 ± 0.00 ^cB^	0.97 ± 0.01 ^aA^
15 min		0.00 ± 0.00 ^aC^	0.20 ± 0.01 ^bD^	0.00 ± 0.00 ^cD^	0.70 ± 0.01 ^bA^	0.38 ± 0.01 ^cB^
20 min		0.00 ± 0.00 ^aD^	0.06 ± 0.00 ^cC^	3.56 ± 0.03 ^aA^	0.00 ± 0.00 ^cD^	0.42 ± 0.01 ^bB^
UT		**P_22_/%**				
		0 W	100 W	200 W	400 W	800 W
0 min		69.26 ± 0.35 ^aA^	69.26 ± 0.35 ^bA^	69.26 ± 0.35 ^dA^	69.26 ± 0.35 ^bA^	69.26 ± 0.35 ^aA^
5 min		69.26 ± 0.35 ^aB^	69.37 ± 0.37 ^bB^	72.67 ± 1.67 ^abA^	66.32 ± 0.32 ^cC^	69.54 ± 0.66 ^aB^
10 min		69.26 ± 0.35 ^aC^	82.48 ± 1.07 ^aA^	74.51 ± 1.51 ^aB^	75.59 ± 1.59 ^aB^	63.08 ± 1.08 ^bD^
15 min		69.26 ± 0.35 ^aB^	69.86 ± 0.34 ^bB^	72.02 ± 1.02 ^bcA^	69.37 ± 1.37 ^bB^	58.14 ± 0.15 ^cC^
20 min		69.26 ± 0.35 ^aA^	66.07 ± 0.54 ^cC^	69.92 ± 0.92 ^cdA^	67.81 ± 0.82 ^bcB^	56.74 ± 0.34 ^dD^
UT		**P_23_/%**				
		0 W	100 W	200 W	400 W	800 W
0 min		30.30 ± 0.03 ^aA^	30.30 ± 0.03 ^aA^	30.30 ± 0.13 ^aA^	30.30 ± 0.33 ^aA^	30.30 ± 0.33 ^cA^
5 min		30.30 ± 0.03 ^aA^	26.67 ± 0.22 ^cC^	23.96 ± 0.97 ^dD^	29.92 ± 0.22 ^abA^	27.95 ± 0.79 ^dB^
10 min		30.30 ± 0.03 ^aB^	14.39 ± 0.11 ^eE^	23.74 ± 0.87 ^dC^	20.88 ± 0.34 ^dD^	32.30 ± 1.22 ^bA^
15 min		30.30 ± 0.03 ^aB^	24.73 ± 0.20 ^dD^	25.29 ± 0.29 ^cD^	27.22 ± 0.56 ^cC^	38.68 ± 0.22 ^aA^
20 min		30.30 ± 0.03 ^aB^	29.66 ± 0.16 ^bBC^	26.53 ± 0.53 ^bD^	29.30 ± 0.34 ^bC^	39.45 ± 0.45 ^aA^

Note: Mean ± standard deviation (n = 3). Lowercase superscript letters (a–d) within the same column indicate statistically significant differences (*p* < 0.05), while uppercase superscript letters (A–D) within the same row denote significant differences (*p* < 0.05). UT: Ultrasonic time UP: Ultrasonic power. 0 min/w: untreated control.

## Data Availability

The original contributions presented in this study are included in the article. Further inquiries can be directed to the corresponding authors.

## References

[1] Xu J., Han L., Yin W. (2022). Research on the ecologicalization efficiency of mariculture industry in China and its influencing factors. Mar. Policy.

[2] Yi X., Pei Z., Xia G., Liu Z., Shi H., Shen X. (2023). Interaction between liposome and myofibrillar protein in surimi: Effect on gel structure and digestive characteristics. Int. J. Biol. Macromol..

[3] Jiao X., Yang H., Li X., Cao H., Zhang N., Yan B., Hu B., Huang J., Zhao J., Zhang H. (2023). Green and sustainable microwave processing of surimi seafoods: A review of protein component interactions, mechanisms, and industrial applications. Trends Food Sci. Technol..

[4] Wang Y., Wang D., Liu J., Yu X. (2023). Effects of rice bran feruloyl oligosaccharides on gel properties and microstructure of grass carp surimi. Food Chem..

[5] Zhang Y., Chen H., Wang X., Li P., Zhao L. (2023). Lipid oxidation-induced protein degradation in surimi gels: Effects of ultrasonic power and duration. Food Chem..

[6] Zhao K., Liu W., Sun T., Zhang Q., Hu F. (2022). Cryoprotectant-free surimi production: Ultrasound-assisted preservation of myofibrillar protein functionality in *Hypomesus olidus*. LWT-Food Sci. Technol..

[7] Wang L., Zhang R., Zhou Y., Chen X. (2023). Time-dependent ultrasonic effects on surimi gel microstructure: A SEM and CLSM study. Food Res. Int..

[8] Guo R., Wu Z., Shi H., Pan Z. (2024). Synergistic effects of ultrasound and ε-polylysine on surimi gel strength: Focus on protein-water interactions. Ultrason. Sonochem..

[9] Jia R., Feng J., Xue Y., Deng S. (2022). Mathematical modeling of ultrasound-assisted surimi gelation: Kinetic analysis of temperature and power effects. J. Food Eng..

[10] Cho S.H., Sohn W.M., Shin S.S., Song H.J., Choi T.G. (2006). Infection status of pond smelts, Hypomesus olidus, and other freshwater fishes with trematode metacercariae in 6 large lakes. Korean J. Parasitol..

[11] Fu Y., Liu C., Yan X., Jiang G., Dang Q., Wang L., Liu X. (2022). Physicochemical and functional properties of the muscle protein fraction of *Hypomesus olidus*. Food Chem. X.

[12] Li K., Fu L., Zhao Y.Y., Xue S.W., Wang P., Xu X.L. (2020). Use of high-intensity ultrasound to improve emulsifying properties of chicken myofibrillar protein and enhance the rheological properties and stability of the emulsion. Food Hydrocoll..

[13] Knorr D., Zenker M., Heinz V., Lee D. (2004). Applications and potential of ultrasonics in food processing. Trends Food Sci. Technol..

[14] McClements D. (1995). Advances in the application of ultrasound in food analysis and processing. Trends Food Sci. Technol..

[15] Soria A., Villamiel M. (2010). Effect of ultrasound on the technological properties and bioactivity of food: A review. Trends Food Sci. Technol..

[16] Bao G., Niu J., Li S., Zhang L., Luo Y. (2021). Effects of ultrasound pretreatment on the quality, nutrients and volatile compounds of dry-cured yak meat. Ultrason. Sonochem..

[17] Cheng Y., Donkor P.O., Ren X., Wu J., Agyemang K., Ayim I., Ma H. (2019). Effect of ultrasound pretreatment with mono-frequency and simultaneous dual frequency on the mechanical properties and microstructure of whey protein emulsion gels—Sciencedirect. Food Hydrocoll..

[18] Gao X., Yongsawatdigul J., Wu R., You J., Xiong S., Du H., Liu R. (2021). Effect of ultrasound pre-treatment modes on gelation properties of silver carp surimi. LWT-Food Sci. Technol..

[19] Li J., Dai Z., Chen Z., Hao Y., Wang S., Mao X. (2022). Improved gelling and emulsifying properties of myofibrillar protein from frozen shrimp (*Litopenaeus vannamei*) by high-intensity ultrasound. Food Hydrocoll..

[20] Chen X., Zhang Y., Wang L. (2023). Ultrasonic modification of food proteins: Mechanisms, applications and challenges. Trends Food Sci. Technol..

[21] Li J., Wang H., Zhao Y. (2022). Effects of ultrasound on the gel properties of aquatic proteins: A review. Food Chem..

[22] Zhao Y., Li J., Wang H. (2023). Advances in ultrasound processing of muscle foods: From mechanism to industrial application. Crit. Rev. Food Sci. Nutr..

[23] Liang F., Lin L., He T., Zhou X., Jiang S., Lu J. (2020). Effect of transglutaminase on gel properties of surimi and precocious chinese mitten crab (*Eriocheir sinensis*) meat. Food Hydrocoll..

[24] Zhang Y., Chen X., Wang L. (2021). Mechanisms of ultrasound-improved gel properties for myofibrillar proteins from silver carp. Ultrason. Sonochem..

[25] Wang L., Chen X., Zhang Y. (2022). Optimization of ultrasound-assisted gelation for improved texture properties of fish protein gels. Food Hydrocoll..

[26] Huang J., Ye B., Wang W., Li J., Mi H. (2021). Incorporation effect of inulin and microbial transglutaminase on the gel properties of silver carp (*Hypophthalmichthys molitrix*) surimi. J. Food Meas. Charact..

[27] Mi H., Li Y., Wang C., Yi S., Li J. (2020). The interaction of starch-gums and their effect on gel properties and protein conformation of silver carp surimi. Food Hydrocoll..

[28] Chen W., Ma H., Wang Y. (2022). Recent advances in modified food proteins by high intensity ultrasound for enhancing functionality: Potential mechanisms, combination with other methods, equipment innovations and future directions. Ultrason. Sonochem..

[29] Zhang F. (2021). Effect of ultrasound-assisted immersion thawing on emulsifying and gelling properties of chicken myofibrillar protein. LWT-Food Sci. Technol..

[30] Sun Y., Ma L., Ma M., Zheng H., Zhang X., Cai L. (2018). Texture characteristics of chilled prepared mandarin fish (*Siniperca chuatsi*) during storage. Int. J. Food Prop..

[31] Jimenez-Munoz L., Quintanilla M., Filomena A. (2019). Managing the lionfish: Influence of high intensity ultrasound and binders on textural and sensory properties of lionfish (*Pterois volitans*) surimi patties. J. Food Sci. Technol..

[32] Li J., Zhang K., Zhao M. (2023). Comparative study of ultrasonic effects on textural properties of cod and carp surimi gels. J. Food Eng..

[33] Zhao M., Jiang H., Chen Q. (2023). Hydrogen bond stability in ultrasonicated Pangasius surimi gels. Food Res. Int..

[34] Chen X., Zhang Y., Wang L. (2023). Species-specific effects of ultrasonication on myofibrillar protein denaturation kinetics. Food Chem..

[35] Jiang H., Wang X., Chen Q. (2022). Microstructure-texture relationships in protein gels: A quantitative SEM-NMR approach. Food Hydrocolloids.

[36] He Z., Liu J., Li Y. (2021). Protein denaturation window hypothesis in ultrasonic processing of aquatic proteins. Ultrason. Sonochem..

[37] Bertram H., Kristensen M., Andersen H. (2004). Functionality of myofibrillar proteins as affected by ph, ionic strength and heat treatment—A low-field nmr study. Meat Sci..

[38] Liu R., Wu L., Zhang Y., Zhang H., Zhang B., Huang B., Wei Y. (2015). Water state and distribution in noodle dough using low-field nuclear magnetic resonance and differential scanning calorimetric. Trans. Chin. Soc. Agric. Eng..

[39] Shi H., Zhang X., Chen X., Fang R., Zou Y., Wang D. (2020). How ultrasound combined with potassium alginate marination tenderizes old chicken breast meat: Possible mechanisms from tissue to protein. Food Chem..

[40] Zhao Y., Wang L., Chen X. (2023). Emerging ultrasonic applications in muscle food processing: Mechanisms and optimization. Trends Food Sci. Technol..

[41] Chen X., Zhang Y., Li J. (2023). Multiscale analysis of ultrasonic effects on protein-water interactions in surimi gels. Ultrason. Sonochem..

[42] Cao Y., Xia T., Zhou G., Xu X. (2012). The mechanism of high pressure-induced gels of rabbit myosin. Innov. Food Sci. Emerg. Technol..

[43] Feng X., Bansal N., Yang H. (2016). Fish gelatin combined with chitosan coating inhibits myofibril degradation of golden pomfret (*Trachinotus blochii*) fillet during cold storage. Food Chem..

[44] Gouda M., Shisi Z., Yuanyuan L., Sheng L., Ma M. (2017). Effects of four natural antioxidant phenyl terpenes on emulsifying and rheological properties of egg yolk. LWT-Food Sci. Technol..

[45] Erturk Y., Bonilla C., Kokini J. (2021). Relationship of non-linear rheological properties and quantitative network analysis parameters as a function of increasingly large amplitude deformations in non-fat, low-fat and high-fat yogurt products. Food Hydrocoll..

[46] Long S., Peishan L., Huiqing W., Yuanyuan L., Ke H., Mostafa G. (2018). Tapioca starch-pullulan interaction during gelation and retrogradation. LWT-Food Sci. Technol..

[47] Wen Y., Yao T., Xu Y., Corke H., Sui Z. (2020). Pasting, thermal and rheological properties of octenylsuccinylate modified starches from diverse small granule starches differing in amylose content. J. Cereal Sci..

[48] Zhu L., Xu Q., Liu X., Xu Y., Yang L., Wang S. (2020). Soy glycinin-soyasaponin mixtures at oil–water interface: Interfacial behavior and o/w emulsion stability. Food Chem..

